# Zinsser-Engman-Cole Syndrome Presenting as Partial Limbal Stem Cell Deficiency

**DOI:** 10.7759/cureus.10933

**Published:** 2020-10-13

**Authors:** Arjun Srirampur, Tarannum Mansoori, Pravalika Rebbala

**Affiliations:** 1 Department of Cornea, Anand Eye Institute, Hyderabad, IND; 2 Department of Glaucoma, Anand Eye Institute, Hyderabad, IND

**Keywords:** dyskeratosis congenita, telomere, limbal stem cell deficiency

## Abstract

Purpose: To report a case of partial limbal stem cell deficiency and characteristic mucocutaneous triad in an 11-year-old boy.

Methods: Systemic features along with ocular features are described in this case report.

Results: Ophthalmic evaluation showed bilateral partial limbal stem cell deficiency (LSCD) and systemic examination revealed characteristic mucocutaneous triad of oral leukoplakia, skin hypopigmentation, and nail dystrophy suggestive of the Zinsser-Engman-Cole syndrome or dyskeratosis congenita.

Conclusions: Thorough ocular and systemic evaluation can help in clinching the diagnosis of this rare condition. Timely referral to a dermatologist and hemato-oncologist would help in the management of life-threatening complications like malignancy or progressive bone marrow failure.

## Introduction

Zinsser-Engman-Cole syndrome, or dyskeratosis congenita (DC), is an inherited, rare progressive congenital disorder characterized by the mucocutaneous triad of abnormal skin pigmentation, nail dystrophy, and oral mucosal leukoplakia [1]. Early diagnosis is important, as serious complications such as malignancy and progressive bone marrow failure can occur. Clinical abnormalities which are first detected in childhood are skin pigmentation and nail changes. DC is primarily a disease of defective telomere maintenance [2]. Telomere function is critically important in humans as its deficiency results in multiple abnormalities including premature aging and cancer. Thus, patients with DC may also show signs of premature aging, especially in the tissues that require constant renewal. Defects in the DNA repair that are linked to the cellular processes with premature aging and carcinogenesis may also affect the eye [3].

Ocular manifestations in DC include epiphora secondary to nasolacrimal duct obstruction, sicca syndrome, conjunctivitis, blepharitis, madarosis, microphthalmia, strabismus, cataract, and retinal vasculopathy [4]. There are very few reports of limbal stem cell deficiency in patients with DC [5].

We report a case of a young boy, who had come for a routine eye examination and on extensive systemic evaluation, he was noted to have bilateral partial limbal stem cell deficiency (LSCD) and characteristic mucocutaneous triad, suggestive of the clinical diagnosis of DC.

## Case presentation

**Figure 1:**
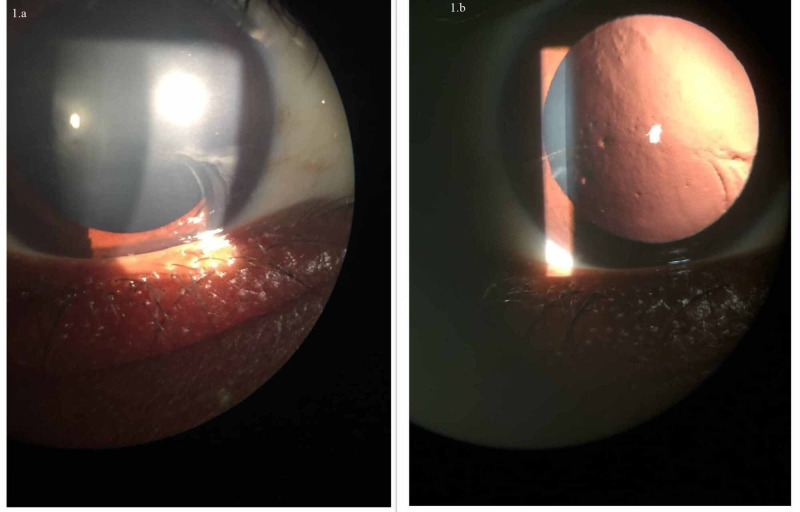
Slitlamp image of the eye Corneal epithelial irregularity and subepithelial scarring better seen on retro illumination.

A 15-year-old boy presented with the complaint of burning sensation in both eyes for four months. There was history of second degree consanguineous marriage in the parents, but no other family member or siblings had a similar complaint. Genetic testing could not be performed. On examination, his best-corrected visual acuity was 20/30 in both eyes. The adnexa and conjunctiva were normal. Both corneas showed superficial punctate keratitis, more marked near the area of the depigmented limbus. The depigmented limbus was suggestive of loss of pigmentation of the limbal palisades of Vogt. Infero-temporally there was a linear 3x1 mm nebular opacity with epithelial cell migration from the adjacent limbus towards the opacity, suggestive of an unhealthy epithelium as seen in Figure 1a.

Fluorescein staining showed granular staining and vortex pattern staining near the area of epithelial cell migration. The image showing the fluorescein staining pattern is not available. Central cornea showed numerous pinpoint opacities, seen clearly on retro illumination as seen in Figure 1b. Lens was clear and intraocular pressure and fundus examination were normal in both eyes.

**Figure 2:**
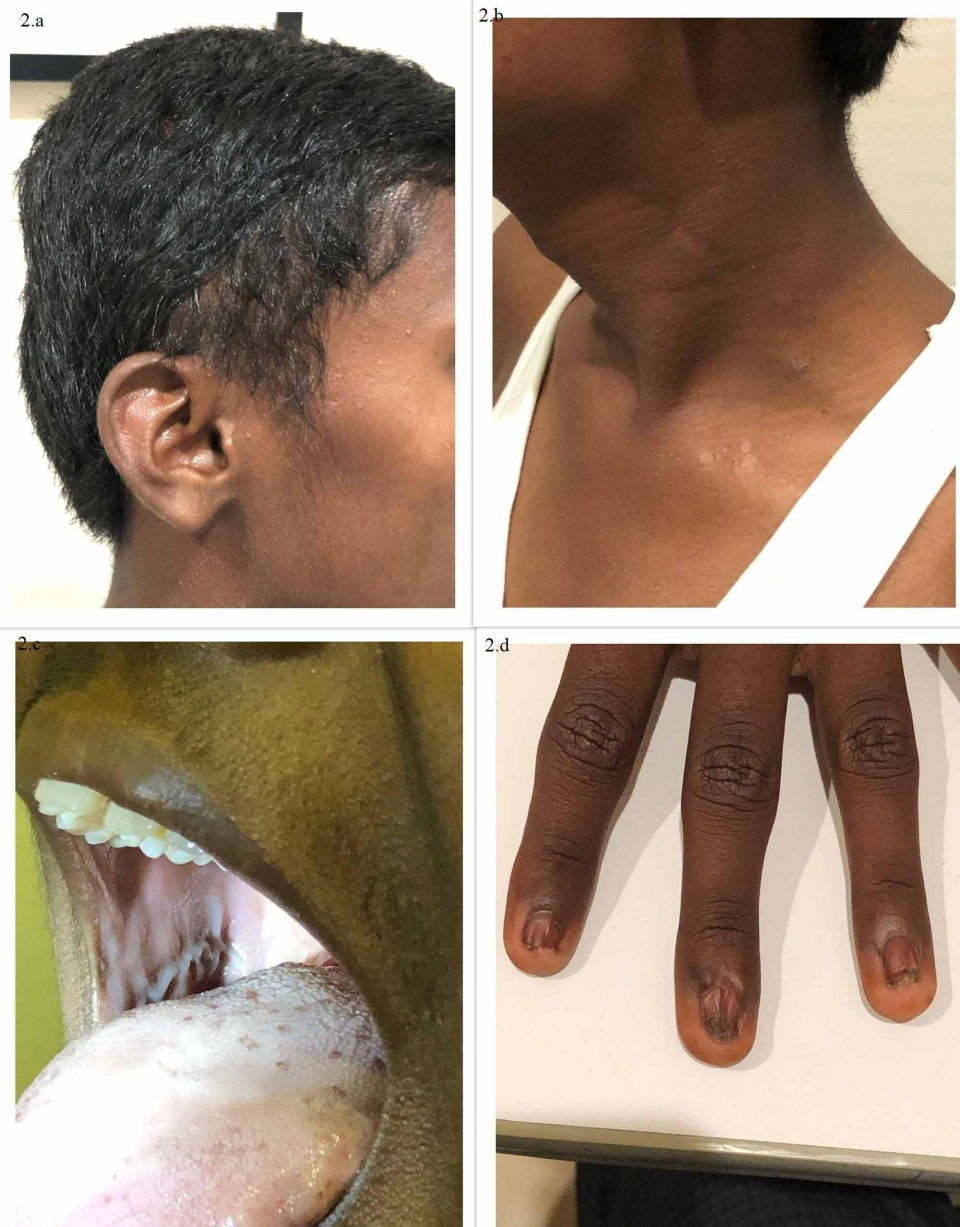
Images showing systemic involvement in the patient Systemic evaluation showing sparse scalp hair (2a), abnormal skin pigmentation (2b), oral leucoplakia (2c), nail dystrophy (2d).

On general examination, the child was of moderate build and anaemic. Systemic evaluation showed sparse hair on the scalp at the temple as seen in Figure 2a. There were multiple small hypopigmented macules on the skin of the neck and trunk as seen in Figure 2b. Oral leukoplakia on the tongue and buccal cavity were noted as seen in Figure 2c. All fingernails and toenails showed rudimentary nails with brown discolouration and dystrophic changes as seen in Figure 2d along with hyperhidrosis of the palms and soles.

Based on the clinical features a clinical diagnosis of DC was made. He was prescribed topical carboxymethylcellulose 0.5% eye drops, one drop six times a day in both eyes. He was advised a complete blood picture, which showed moderate to severe anemia (hemoglobin 6 g/dL) and severe thrombocytosis (platelets >8,000,000/mm3). He was referred to the dermatologist and hematologist for further management.

## Discussion

Dyskeratosis congenita (DC), initially described as a bone marrow failure syndrome, is a multisystem disorder with a very broad range of clinical presentations. Classically, the disease is defined by a triad of mucocutaneous features: nail dystrophy, oral leukoplakia, and abnormal skin pigmentation [1]. A wide spectrum of disorders affecting every system in the body, including the eyes, has been described in this disease. Since various ocular manifestations have been observed in genomic instability syndromes, it is considered that this pathology has developed related to DC rather than a coincidence. Telomeres play a central role in cell turnover and aging, and their length can directly affect the ability of stem cells to regenerate tissues [2].

Limbal epithelial stem cells are located in the basal epithelial layer of the limbus (corneo-scleral junction). Characteristics of limbal stem cells include a slow turnover rate, high proliferative potential, clonogenicity, expression of stem cell markers, as well as the ability to regenerate the entire corneal epithelium in normal physiological and pathological conditions [5]. Premature shortening of telomeres results in a limited proliferative potential of these limbal stem cells and consequently premature aging which leads to LSCD. Limbal stem cell deficiency has been stated as a manifestation of stem cell deficiency in DC [6]. Impression cytology and confocal microscopy of the corneal epithelium can be performed to confirm the diagnosis of LSCD.

Our patient presented to the clinic for a routine ocular examination and on systemic evaluation he was noted to have the classical triad of dermatologic, oral, and hair abnormalities along with partial LSCD that suggested a clinical diagnosis of DC. Similar presentations of DC with LSCD and systemic abnormalities have been described previously in the literature [5].

The diagnosis of LSCD in this case was based on clinical features. We could not perform confocal microscopy or impression cytology to confirm the diagnosis of LSCD. Appropriate genetic testing to confirm the disease was not done, due to the financial constraints of the patient. Confirmation with a genetic test would add more value and insight in managing such cases. 

## Conclusions

In conclusion, as an ophthalmologist one should be aware of this rare and life-threatening condition as the patient may seek help for eye problems secondary to the LSCD and we can play a major role in recognizing the case. Failure to recognize the condition can prove to be fatal due to life-threatening conditions like malignancy or progressive bone marrow failure. A thorough ocular and systemic evaluation would help in clinching the timely diagnosis.
